# Neo-Epitopes Generated on Hydroxyl Radical Modified GlycatedIgG Have Role in Immunopathology of Diabetes Type 2

**DOI:** 10.1371/journal.pone.0169099

**Published:** 2017-01-03

**Authors:** Sidra Islam, Abdul Rouf Mir, Alok Raghav, Farzana Khan, Khursheed Alam, Asif Ali, Moin Uddin

**Affiliations:** 1 Department of Biochemistry, J.N. Medical College, Faculty of Medicine, Aligarh Muslim University, Aligarh, Uttar Pradesh, India; 2 Department of Biotechnology, Government Degree College Baramulla, University of Kashmir, Jammu and Kashmir, India; 3 Rajiv Gandhi Centre for Diabetes and Endocrinology, Aligarh Muslim University, Aligarh, Uttar Pradesh, India; Shiraz University, ISLAMIC REPUBLIC OF IRAN

## Abstract

Glycoxidation plays a crucial role in diabetes and its associated complications. Among the glycoxidation agents, methylglyoxal (MG) is known to have very highglycationpotential witha concomitant generation of reactive oxygen species (ROS) during its synthesis and degradation. The presentstudy probes the MG and ROSinduced structural damage to immunoglobulin G (IgG) and alterations in its immunogenicity in diabetes type 2 patients (T2DM). Human IgG was first glycated with MG followed by hydroxyl radical (OH^•^) modification. Glycoxidation mediated effects on IgG were evaluated by various physicochemical techniques likeultraviolet (UV) and fluorescence spectroscopy, 8-anilinonaphthalene-1-sulfonic acid (ANS) binding studies, carbonyl andfree sulfhydryl groups assay, matrix assisted laser desorption ionization mass spectrometry-time of flight (MALDI-TOF), red blood cell (RBC) haemolysis assay, Congored (CR) staining analysis and scanning electron microscopy (SEM). The results revealed hyperchromicityin UV, advanced glycation end product (AGE)specific and ANS fluorescence, quenching in tyrosine and tryptophan fluorescence intensity,enhanced carbonyl content,reduction in free sulfhydryl groups,pronounced shift in m/z value of IgGand decrease in antioxidant activity in RBC induced haemolysis assayupon glycoxidation. SEM and CRstaining assay showed highly altered surface morphology in glycoxidised sample as compared to the native. Enzyme linked immunosorbent assay (ELISA) and band shift assay were performed to assess the changes in immunogenicity of IgG upon glyoxidation and its role in T2DM. The serum antibodies derived from T2DM patients demonstrated strong affinity towards OH^•^ treated MG glycatedIgG (OH^•^-MG-IgG) when compared to native IgG (N-IgG) or IgGs treated with MG alone (MG-IgG) or OH^•^ alone (OH^•^-IgG). This study shows the cumulating effect of OH^•^ on the glycation potential of MG. The results point towards the modification of IgG in diabetes patients under the effect of glycoxidative stress, leading to the generation of neo-epitopes on theIgG molecule and rendering it immunogenic.

## Introduction

There is an overwhelming literature supporting the indulgence of reactive oxygen species (ROS)and reactive carbonyl species (RCS) in severe pathogenesis of aging, cancer, diabetes and its associated complications[1, 2]. The non-enzymatic synthesis of glycated adducts formed by the reaction of proteins withreducing sugar contribute in the pathogenesis of diabetic complications via free radical generation that promote carbonyl formation, fragmentation and cross linking of proteins[3–5]. Among the sugar derivatives,methylglyoxal (MG) is a reactive dicarbonyl compound having20,000 times more glycatingpotential than glucose[6].It is produced by degeneration of lipid peroxidation products (LPP), autoxidation of sugars, dephosphorylation of polyol pathways and glycolytic intermediates such as glyceraldehyde-3-phosphate (G3P) and dihydroxyacetone phosphate (DHAP) as well as oxidation of hydroxyacetone and aminoacetone[7, 8]. MGreacts with a variety of biological macromolecules forming fluorescent and non-fluorescent crosslinks[8–11].Previous literature has reported that the concentration of MG in diabetes patients increases many folds in lens, blood and kidney [12–15].Adirect link between free radical generation and MG toxicityis well known [16]. ROS production by MG was first described in 1993 and since then, the mutual interdependency between free radicals and MG is widely reported[17].Diabetes patients have elevated plasma MG levels that inactivate antioxidant enzymes and thereby accumulate an oxidative stress[18–21]. MG is a key player in the modification of amino acids,nucleic acids [14, 22] and specific binding of MG modified proteins leads to immunological complications in diabetes patients [10, 15, 23, 24].This work aims to study the hydroxyl radical(OH^•^) mediated structural perturbations in MG glycated immunoglobulin G (IgG) byvarious biophysical and biochemical techniques like ultraviolet (UV) and fluorescence spectroscopy, 8-anilinonaphthalene-1-sulfonic acid (ANS) binding studies, estimation of carbonyl content and free sulfhydryl groups, matrix assisted laser desorption/ionization time-of-flight mass spectrometry (MALDI-TOF MS), red blood cell (RBC)haemolysis assay, congored(CR)staining analysis and scanning electron microscopy(SEM). Furthermore, this work demonstratesthe changes in immunogenicity of IgG upon OH^•^-MG mediatedglycoxidation and its role in the immunopathology of diabetes type 2 (T2DM).

## Materials and Methods

Anti-human alkaline phosphatase conjugate, p-nitrophenyl phosphate (PNPP), tween 20, sodium dodecyl sulphate (SDS), protein-Aagarose affinity column, fruend’scomplete (CFA) and incomplete adjuvant (IFA), sodium azide, agarose and dialysis tubing were obtained from Sigma Chemical Company (U.S.A).Acrylamide, bisacrylamide, ammonium persulfate (APS) and N,N,N’,N’tetraethylenediamine(TEMED) were from qualigens(India) and silver nitrate from SRL (India).

### Clinical sampling

The study was undertaken on T2DM patients (n = 80; age >20 years), excluding those with micro and macro-vascular complications, type 1 diabetes (T1DM) and gestational diabetes (GDM).Healthy subjects (n = 20) of the same age group were takenas control. Blood was taken in clot activator vials and serum was separated by centrifugation at 3000 rpm for 10 min followed by heating at 56°C for 30 min to inactivate complement proteins and stored in aliquots at -20°C with 0.1% sodium azide as preservative [25].

### Ethical statement

The study was approved by institutional ethics committee (certificate approval no. 1297/FM) at J. N. Medical College,AMU, Aligarh.Blood was taken in clot activator vials only after written informed consent from both the patients and healthy individuals and a proper record of all the patients and healthy individuals has been maintained.

### Isolation of IgG

Blood from healthy individuals was obtained and allowed to coagulate at 37°C for 30min. It was centrifuged at 3000 rpm for 10 min to obtain the serum which was heated at 56°C for 30 min to inactivate complement proteins. IgG was isolated by affinity chromatography using Protein-Aagaroseaffinity column and its concentration was determined considering 1.4 O.D._278_ = 1mg/ml IgG[25].Homogeneity of the IgGwas checked on 7.5% SDS-PAGE and it was stored at -20°C with 0.1% sodium azide as preservative.

### Preparation of OH^•^treated MG glycatedIgG (OH^•^-MG-IgG)

IgG (1μM) was incubated under sterile conditions with MG (7.5 mM) in phosphatebuffer saline (PBS) (10 mM, pH 7.4) at 37°C for 24 hours. It was followed by OH^•^modification (30 min) of theglycated sample. The OH^•^ radicalwas generated by fenton’s reaction. Assay tubes containing glycatedIgGwere incubated with 100 μM hydrogen peroxide (H_2_O_2_) and 30 μM FeCl_2_/EDTAfor half an hour at 37°C. The reaction was then stopped by using 0.1% trifluoroacetic acid (TFA)[4]. The effect of OH^•^-MG on IgG was studied with IgG alone, MG modified IgG (MG-IgG)and OH^•^ modified IgG (OH^•^-IgG)as control samples. The samples were extensively dialyzed against sterile PBS (10mM, pH 7.4)and stored at -20°C until further analysis.

### UV-Absorption spectroscopy

UV absorption spectra of native and modified IgG were recorded in 250–400 nm wavelengthrange onShimadzu UV-1700 spectrophotometer [26]. Hyperchromicity at 280 nmwascalculated by following equation:

$$\%\text{ Increase in hyperchromicity at }280\text{nm}=\frac{{\text{OD }}_{\text{ modified IgG}}-{\text{OD}}_{\text{native IgG}}}{{\text{OD}}_{\text{ modified IgG}}}\times 100$$ (1)

### Fluorescence spectroscopy

To detect structural changes in the modified IgG, fluorescence spectra were taken on Shimadzu (RF-5301-PC) spectrofluorometerat 25±0.2°C using quartz cuvette of 1 cm path length. The fluorescence intensities of tyrosine and tryptophan were monitored by exciting the samples at 275 and 295 nm and the emission spectra were recorded in the range of 280–400 and 300–400 nm respectively [27]. Decrease in fluorescence intensities in caseof modified samples were calculated using the following equation

$$\%\text{ loss in flourescence intensity}=\frac{{\text{FI}}_{\text{native IgG}}-{\text{FI}}_{\text{ modified IgG}}}{{\text{FI}}_{\text{native IgG}}}\text{ X }100$$ (2)

AGE-specific fluorescence was also monitored by exciting the samples at 370 nm and recording emission spectra in the 380–550 nm range[28]. Increase in fluorescenceintensity was calculated using the following equation

$$\%\text{ increase in flourescence intensity}=\frac{{\text{FI}}_{\text{modified IgG}}-{\text{FI}}_{\text{native IgG}}}{{\text{FI}}_{\text{modified IgG}}}\text{ X }100$$ (3)

### ANS binding studies

The conformational changes in the modified IgG were further evaluated byANS binding and recorded in terms of fluorescence.TheIgG to ANS molar ratio was 1:50 and the emission spectra were recorded in the wavelength range of 400–600 nm after exciting the samples at 380 nm[29].Increase in fluorescence intensity was calculated as given below:

$$\%\text{ increase in fluorescence intenisty}=\frac{{\text{FI }}_{\text{modified IgG}}-{\text{FI}}_{\text{native IgG}}}{{\text{FI}}_{\text{modified IgG}}}\times 100$$ (4)

### Carbonyl content

Carbonyl content of native and modified IgG wasquantitated as per the published protocol [30].

### Free sulfhydryl group content

Free sulfhydryl group contents of native and modified IgG were determined as per the established method[31].

### MALDI-TOF studies

MALDI-TOF MSwas performed on 4800 plus MALDI-TOF mass spectrometer operating in a positive ion mode. 0.95 μl of 1 mg/ml samplewas spotted on a 384 well insert opti-TOF-stainless steel MALDI plate. Sinapinic acid dissolved in 10 mg/ml acetonitrile and 0.1% TFA was used as a matrix. Analysis was carried out using protein chip software.

### RBC haemolysis assay

The antioxidant characteristics of native and modified IgG were evaluated by RBC haemolysis assay[32]. Healthy human blood samples from the J.N. Medical College were obtained in EDTA vials. Erythrocytes from the plasma were isolated and washed three times with isotonic saline (NaCl, 0.15 M), and centrifuged at 4000 rpm for 10 minutes at 4°C. 100 μl (approx 1X 10^8^ erythrocytes, 400,000 cells/μl final concentrations) of diluted RBC (1/10 in 0.15 M NaCl) were added into each well of flat bottom 96 well-plate. 10 μM each of nativeIgG(N-IgG), OH^•^-IgG,MG-IgG andOH^•^-MG-IgGwere then added to the well in duplicate. This was followed by addition of 20 μl of 200 mMof 2,2'-Azobis(2-amidinopropane) dihydrochloride (AAPH), a peroxyl radical initiator. Diluted RBC alone and RBC with AAPH were taken as positive and negative controls respectively. The samples were incubated at 37°C and absorbance was recorded on microplate reader at 540 nm every 20 min until constant readings were achieved in each well. Results are expressed as time (in minutes) required for 50% of maximal inhibition in haemolysis of RBCs (H_T 50_) using the following equation [33].

$$\%\text{ Inhibition}=\frac{{\text{A }}_{\text{AAPH}}-{\text{A}}_{\text{sample}}}{{\text{A}}_{\text{AAPH}}}$$ (5)

A_AAPH_ = Absorbance of AAPH at 540 nm

A_sample_is the absorbance of IgG (native and modified) at 540 nm.

### Spectacle crosslink’s in modified IgG

CR staining was done to study aggregation as a result of protein (IgG) modification. Staining was done as per previously published procedure [34].Briefly, 200–400 μl of freshly prepared CR solution was put on slides having 10 μl of air dried IgG (5 μM). The slides were then examined under polarised light microscope. The detection of yellow or green colour birefringenceindicates the presence of the protein aggregates in the samples. Images of the stained slides were recorded with the camera attached to the microscope.

### Scanning electron microscopy of modified IgG

SEM imaging was used to analyze changes in micro-architecture of the protein upon modification. 20 μl of 5 μMsampleswere air-dried and then adsorbed on cellulose ultra filtrationmembrane.The samples were then coated with gold and mounted overstainless steel grids covered with collodion film of carbon operating under low vacuum at 15kV. The samples were imaged under JSM-6510LV (JEOL JAPAN) scanning electron microscope at a 1000X resolution [35, 36].

### Direct binding ELISA

ELISA was performed on flat bottom polystyrene plates as described earlier [37]. Briefly, 96 wells polystyrene polysorpimmunoplates were coated with 100μl of N-IgG, MG-IgG, OH^•^-IgGand OH^•^-MG-IgG(10 μg/ml) in antigen coating buffer. The plates were incubated for 2 hr at 37°C followed by overnight incubation at 4°C. Each sample was coated in duplicate and half of the plate, devoid of antigen coating, served as control. The plates were washed 3 times with tris buffered saline containing tween 20 (TBS-T) and unoccupied sites were blocked with 150 μl of 2.5% `fat free skimmed milk in tris buffered saline (TBS) for 4–5 hr at 37°C. After incubation, the plates were washed thrice with TBS-T and coated for 2 hrwith sera or affinity purified IgG from diabetes patients. Evaluation of bound antibodies was done using anti-human IgG alkaline phosphatase conjugate using PNPP as substrate. Absorbance (A) of each well was monitored at 410 nm on an automatic microplate reader and the mean of duplicate readings for each sample was recorded. Results have been expressed as a mean ofA_test_—A_control_.

### Inhibition ELISA

The antigenic specificity of antibodies was determined by competitive inhibition ELISA [38].Varying amounts of inhibitors (0–20 μg/ml) were mixed with a constant amount of antiserum or affinity purified IgG and the mixture was incubated for 2 hrat 37°C and overnight at 4°C. The immune complex thus formed was loadedin the antigen coated wells instead of the serum. The remaining steps were the same as in direct binding ELISA. Percent inhibition was calculated using the formula:

$$\text{Percent inhibition}=1-\frac{{\text{A }}_{\text{inhibited}}}{{\text{A}}_{\text{uninhibited}}}\times 100$$ (6)

### Band shift assay

For the visual detection of antigen–antibody binding, gel retardation assay was performed [39]. Immune complexes were prepared by incubating constant amount of native/modified antigen with varying amounts of affinity purified IgG in PBS for 2hr at 37°C and overnight at 4°C. One-fourth volume of sample dye was added to the mixture and electrophoresed on 6% SDS-polyacrylamide gel for 3 hr at 80 V. The gels were visualized by staining with silver nitrate.

### Statistical analysis

Data are presented as mean±SD. Statistical significance of the data was determined by Student’s *t*test, and a value of *p* < 0.05 was considered as statistically significant.

## Results

### UV absorbance spectroscopy

**Fig 1:**
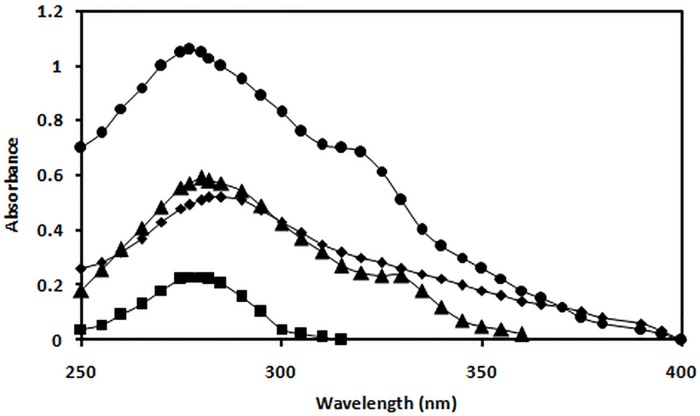
Absorbance study. UV absorption spectra of N-IgG(■), OH^•^-IgG (◆), MG-IgG (▲) and OH^•^-MG-IgG (●).

The absorbance values at 280 nm for N-IgG, OH^•^-IgG, MG-IgG and OH^•^-MG-IgG wereobserved to be 0.230, 0.522, 0.652 and 1.06respectively. The data analysis showed 78.3%, 64.7%, 55.9% hyperchromicityfor OH^•^-MG-IgG, MG-IgG and OH^•^-IgG samples compared respectively to the N-IgG. A hump like structure at 330 nm was observed in MG-IgG and OH^•^-MG-IgG(Fig 1).

### Intrinsic fluorescence assay

**Fig 2:**
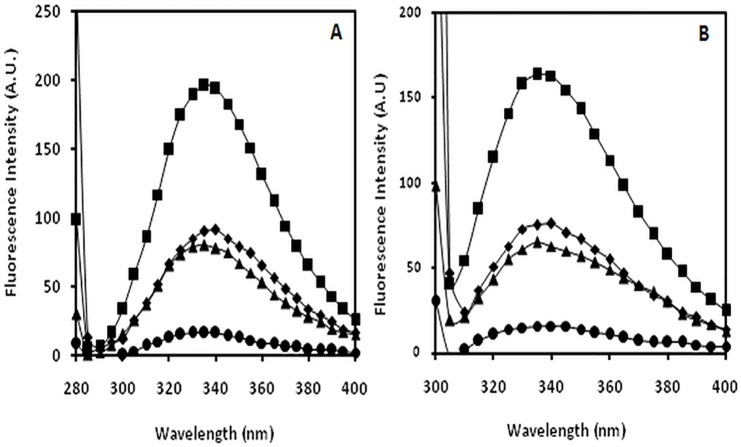
Fluorescence study. Fluorescence profile of N-IgG (■), OH^•^-IgG (◆), MG-IgG (▲)and OH^•^-MG-IgG(●) A) excitation at 280 nm (tyrosine intrinsic fluorescence intensity) B) excitation at 295 nm (tryptophan intrinsic fluorescence intensity).

Fluorophoric properties of tyrosine and tryptophan amino acid residues were utilised to record changes incurred upon modification. The observed tyrosine intrinsic fluorescence intensities for N-IgG, OH^•^-IgG, MG-IgG and OH^•^-MG-IgG were observed to be 196.15, 90.00, 80.19 and 19.61 respectively. The data analysis showed decrease in tyrosine intrinsic fluorescence intensity by 54.1%, 59.1% and 90.0% for OH^•^-IgG, MG-IgG and OH^•^-MG-IgG samples respectively, when compared to N-IgG. The observed tryptophan intrinsic fluorescence intensities for N-IgG, OH^•^-IgG, MG-IgG and OH^•^-MG-IgG were observed to be 163.49, 76.00, 65.01 and 14.71 respectively. The data analysis showed decrease in tryptophan intrinsic fluorescence intensity by 53.5%, 60.2% and 91.0%for OH^•^-IgG, MG-IgG and OH^•^-MG-IgGsamplescompared to that of N-IgG respectively (Fig 2).

### AGE specific fluorescence

**Fig 3:**
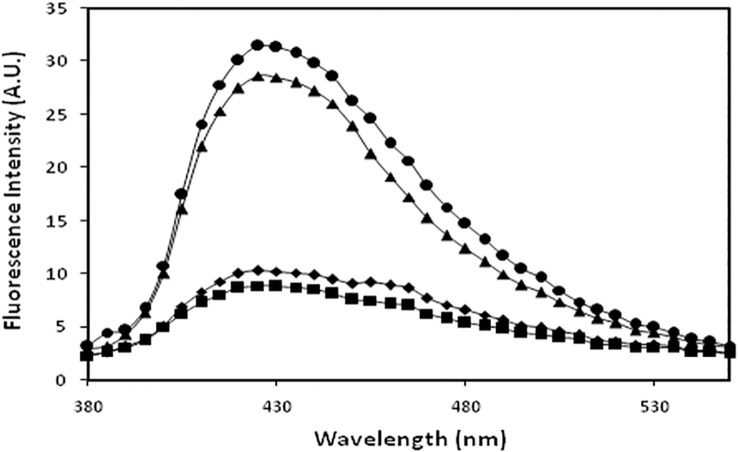
AGE assay. AGE specific fluorescence profile of N-IgG (■), OH^•^-IgG (◆), MG-IgG (▲) and OH^•^ -MG- IgG (●).

Fluorescence spectroscopy was also used to detect formation of AGEs by exciting samples at 370 nm with emission spectra recorded in the range of 380–550 nm. N-IgG and OH^•^-IgGexhibited negligible fluorescence at 370 nm excitation indicating no fluorogenic AGEs. MG-IgG and OH^•^-MG-IgG showed 69.1% and 71.0% hyperchromicity compared to native protein underidentical experimental conditions (Fig 3).

### Determination of surface hydrophobicity using ANS dye

**Fig 4:**
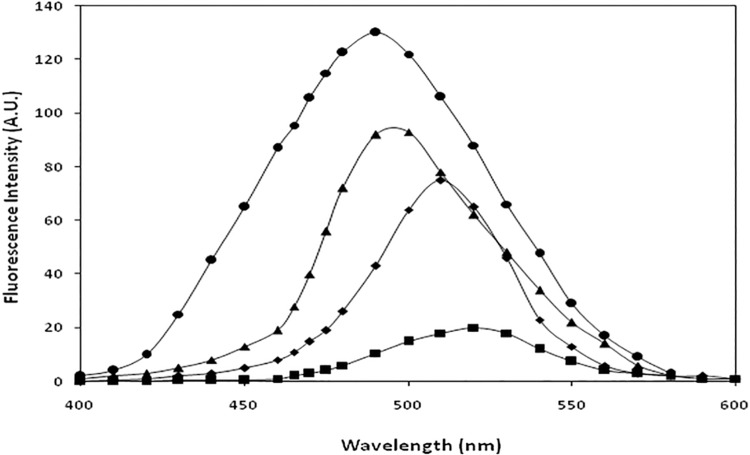
Relative ANS fluorescence. ANS binding fluorescence profile of N-IgG (■),OH^•^ -IgG(◆), MG-IgG(▲) andOH^•^-MG-IgG(●).

A significant decrease in the ANS fluorescence intensity was observed in IgG upon modification. N-IgG, OH^•^-IgG, MG-IgG and OH^•^-MG-IgG showed ANS binding fluorescence intensities as 20.21, 75.16, 93.24 and 130.01respectively. The data analysis showed 73.1%, 78.3% and 84.4% increase in ANS fluorescence intensity for OH^•^-IgG, MG-IgG and OH^•^-MG-IgGsamples compared to that of N-IgG respectively.FurtherOH^•^-IgG, MG-IgG and OH^•^-MG-IgG showed a blue shift of 9 nm, 21 nm and 29 nm respectivelyin comparison to that ofN-IgG (Fig 4).

### Reactive carbonyl content

**Fig 5:**
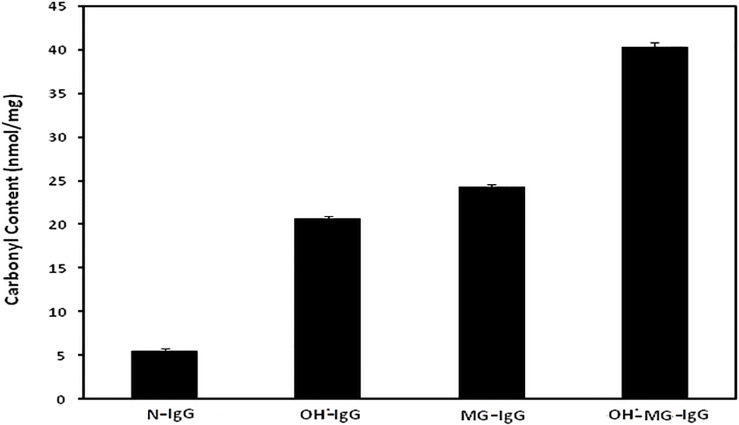
Carbonyl assay. Protein bound carbonyl estimation of N-IgG, OH^•^-IgG, MG-IgG and OH^•^-MG-IgG.

Carbonyl content of N-IgG, OH^•^-IgG, MG-IgG and OH^•^-MG-IgG came out to be 5.5±0.3, 20.6±0.4, 24.2±0.4 and 40.2±0.6 nmol/mg of protein respectively. Each value is average ± SD of3 independent determinations (Fig 5).

### Reactive sulphydryl group content

**Fig 6:**
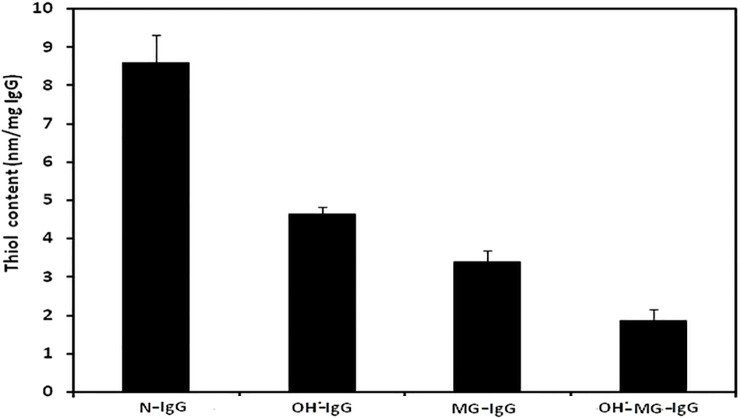
Sulphydryl group assay. Protein boundthiol content of N-IgG, OH^•^-IgG, MG-IgG and OH^•^-MG-IgG.

Reactive sulphydryl group content of N-IgG, OH^•^-IgG, MG-IgG and OH^•^-MG-IgG came out to be 8.6±0.7, 4.6±0.2, 3.4±0.3 and 1.8±0.3 nmol/mg of IgG respectively. Each value is average ± SD of 3independent determinations (Fig 6).

### MALDI-TOF analysis

**Fig 7:**
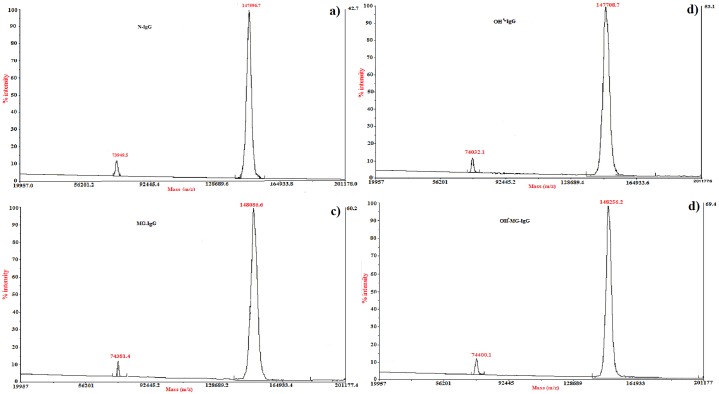
Mass spectroscopic analysis. MALDI-TOFmass spectral profile of N-IgG (a), OH^•^-IgG (b), MG-IgG (c) and OH^•^-MG-IgG (d).

MALDI analysis showed two obvious peaks for N-IgG at m/z ratio of 73949.5 and 147596.7. The m/z mass peaks for OH^•^-IgG were observed at 74032.1 and 147708.7. The m/z ratio for MG-IgG was found to be 74381.4 and 148086.6. The maximum change was observed in the m/z ratio of OH^•^-MG-IgG with values as 74400.1 and 148256.2. Compared to the N-IgG, themass spectrometric analysis corresponded to an incrementin m/z ratio by194.6 Da for OH^•^-IgG, 921.8 Da for MG-IgGand 1110.1 Da for OH^•^-MG-IgGrespectively (Fig 7).

### Aggregation and cross linking studies

**Fig 8:**
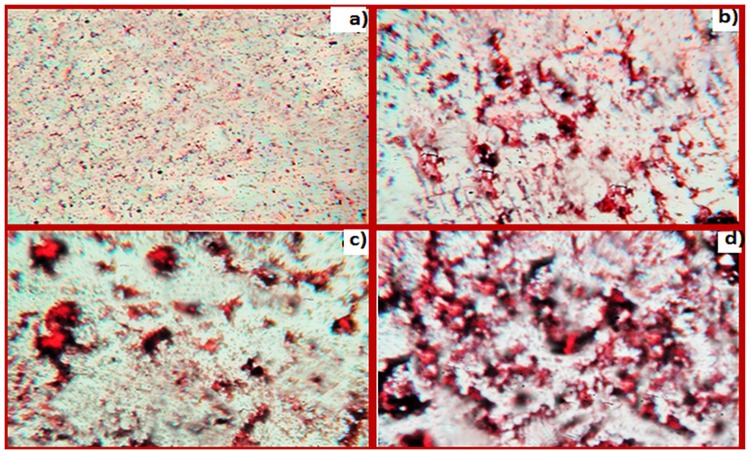
CR staining analysis. CR stained samples of N-IgG (a), OH^•^-IgG (b), MG-IgG (c) and OH^•^-MG-IgG (d). The images were taken at a resolution of 10X.

**Fig 9:**
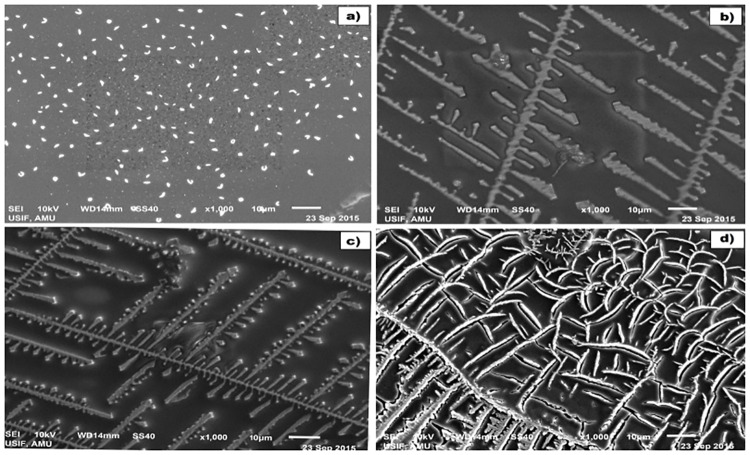
SEM analysis. Imagesof N-IgG (A), OH^•^-IgG (B), MG-IgG (C) and OH^•^-MG-IgG (D).The images were taken at a resolution of 1000X and a scale bar of 10 μm.

Analysis of samples incubated with CR demonstrated significant aggregate formation in OH^•^-MG-IgGsamples compared to OH^•^-IgG, MG-IgGand N-IgG (Fig 8). These results have been further confirmed by SEM wherein modification with OH^•^, MG and OH^•^-MG resulted in the formation of elongated fibril like aggregates. The OH^•^-MG-IgG showed maximum aggregate formation. A remarkable difference was observed in the micrographs of N-IgGcompared to its modified counterparts(Fig 9).

### AAPH-mediated RBC haemolysis assay

**Fig 10:**
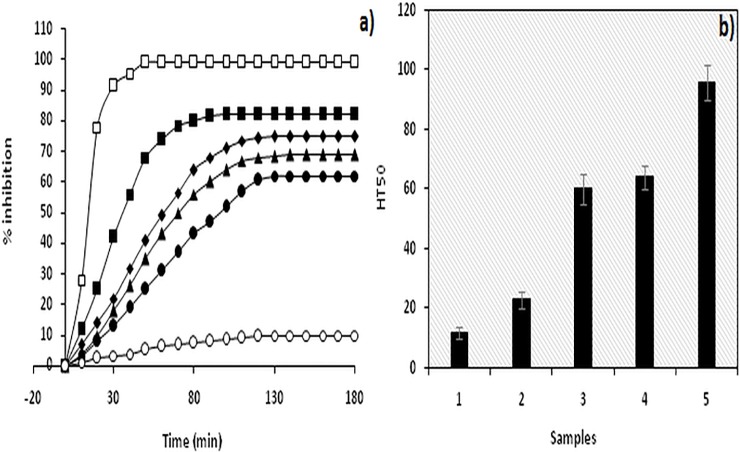
RBC haemolysis assay. AAPH mediated RBC haemolysis assay to evaluate antioxidant status of a) N-IgG (■), OH^•^-IgG(◆), MG-IgG(▲) and OH^•^-MG-IgG(●). RBC alone (□) and RBC incubated with AAPH (o) served as positive and negative control. B) Histogram showing time required for 50% of maximum inhibition in haemolysis (HT_50_) of RBC alone (1),N-IgG (2), OH^•^-IgG (3),MG-IgG (4) and OH^•^-MG-IgG (5). Results are average±SD of 3 independent experiments.

Antioxidant property of proteins is of paramount importance with potential beneficial effects in relieving oxidative stress [40].Owing to typical heart shaped structure and availability of reduced cysteine and methionine residues, the antioxidant property of human serum albumin (HSA) is well established [41].Intrinsic antioxidant capacity of HSA to protect erythrocytes from haemolysis has been evaluated earlier[33]. Using the protocol, we replicated the experiment forIgG(Fig 10). HT_50_ (time required for 50% inhibition in haemolysis) for N-IgG, OH^•^-IgG, MG-IgGand OH^•^-MG-IgG was found to be 23±3 min, 60±5 min, 64±4 min and 96±6 min respectively. Each value is mean ±SDof 3 independent determinations.

### Direct binding ELISA

**Fig 11:**
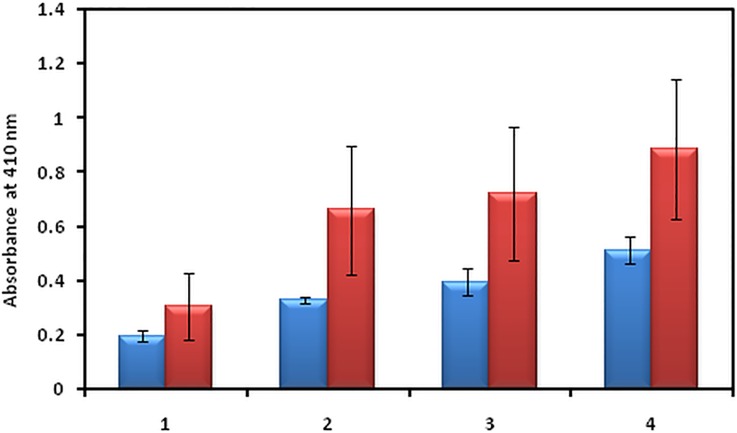
Direct binding ELISA. Serum samples from diabetes patients (red bar) and NHS (blue bar) binding to N-IgG (1), OH^•^-IgG (2), MG-IgG (3) and OH^•^-MG-IgG (4). The results are average±SD of 80 sera from diabetes patients and 20 NHS.

To evaluate the possible cumulative role of MG and OH^•^in the immunopathogenesis of T2DM, 80 serum samples from T2DM patients and 20 normal human subjects were tested for binding to N-IgG, OH^•^-IgG, MG-IgG and OH^•^-MG-IgG. The average absorbance at 410 nm for the binding of serum antibodies to N-IgG, OH^•^-IgG, MG-IgG and OH^•^-MG-IgGwas 0.31±0.12, 0.66±0.24, 0.72±0.24 and 0.89±0.25 respectively.Significantly higher percentage of serum autoantibodies from T2DM patients (66.2%) showed preferential binding to OH^•^-MG-IgG. The binding affinity was followed by MG-IgG and OH^•^-IgGcontrols compared to the N-IgG(Fig 11).

### Inhibition ELISA

Serum samples from diabetes patients showing high binding were further evaluated bycompetitive inhibition ELISA using N-IgG, OH^•^-IgG, MG-IgG and OH^•^-MG-IgG asinhibitors. Maximum percent inhibition was observed when OH^•^-MG-IgGwas taken as inhibitor. The average percent inhibition in the activity of antibodies from diabetes patients by N-IgG, OH^•^-IgG, MG-IgG and OH^•^-MG-IgG came out to be 26.2±5.3, 50.3±4.7, 50.1±2.8 and 64.6±4.1 respectively. Competitive inhibition data of serum antibodies in T2DM patients has been depicted in Table 1.

**Table 1:** Competitive Inhibition ELISA of Serum Antibodies from Diabetes Type 2 Patients.

Sera No	Maximum percent inhibition at 20 μg/ml			
	N-IgG	OH^•^-IgG	MG-IgG	OH^•^-MG-IgG
9	12.4	47.1	52.2	66.4
10	30.3	50.4	55.4	64.9
12	25.2	42.5	47.3	67.3
19	22.2	47.1	52.2	62.6
20	26.4	50.1	48.6	60.6
27	22.2	55.6	51.5	68.2
35	25.9	44.4	51.6	61.9
36	29.0	51.6	48.3	68.0
41	21.6	51.6	49.5	66.3
44	24.4	46.4	55.2	64.9
50	31.7	50.4	46.8	67.4
51	21.7	49.8	48.9	59.1
59	29.2	42.2	45.8	52.5
61	31.5	55.5	49.4	63.4
63	32.4	56.9	50.6	69.1
65	27.4	56.6	51.5	67.1
70	23.6	52.0	45.8	67.6
71	34.7	56.4	50.7	65.4
**Mean±SD**	**26.2±5.3**	**50.3±4.7**	**50.1±2.8**	**64.6±4.1**

The microtitre plates were coated with 10 μg/ml of OH^•^-MG-IgG

### IgG isolation from the sera of diabetes patients

IgG was isolated from 10 serum samples of T2DM patients showing high specificity forOH^•^-MG-IgG[33]. Elute showed a single symmetrical peak at 280 nm and a single band on 7.5% SDS-PAGE confirming the homogeneity of the IgG (data not shown).

### Direct binding ELISA of isolated IgG

Thesaturation in the binding of the patient’s IgG to OH^•^-MG-IgG was evaluated through direct binding ELISA on the microtitre plate coated with OH^•^-MG-IgG. The saturation was achieved at 40 μg/ml of IgG(data not shown).

### Competitive inhibition ELISA of IgG isolated from diabetes patients

Inhibition ELISA of the IgG isolated from the sera of diabetes patients showed OH^•^-MG-IgGas the most powerful inhibitor followed by MG-IgG, OH^•^-IgGand N-IgG. The average percent inhibition of patient’sIgGbinding to N-IgG, OH^•^-IgG, MG-IgG, OH^•^-MG-IgG was found to be 32.4±6.0, 57.5±4.9, 56.1±4.1 and 74.7±3.9 respectively. Table 2 summarizes competitive inhibition data of IgG isolated from T2DM patients.

**Table 2:** Competitive Inhibition ELISA of IgGfrom Diabetes Type 2 Patients.

Sera No.	Maximum percent inhibition at 20 μg/ml			
	N-IgG	OH^•^-IgG	MG-IgG	OH^•^-MG-IgG
9	20.1	52.7	58.7	72.3
12	31.7	50.1	52.1	73.6
20	33.8	57.7	54.4	70.2
32	32.6	51.6	57.9	78.5
36	35.1	59.9	51.6	69.4
51	27.7	63.4	55.7	80.8
63	39.7	64.2	56.7	78.7
65	32.4	57.1	65.5	76.6
69	30.1	56.3	52.8	71.5
71	41.7	62.2	56.3	75.4
**Mean±SD**	**32.4±6.0**	**57.5±4.9**	**56.1±4.1**	**74.7±3.9**

The microtitre plates were coated with 10 μg/ml of OH^•^-MG-IgG

### Gel retardation assay

**Fig 12:**
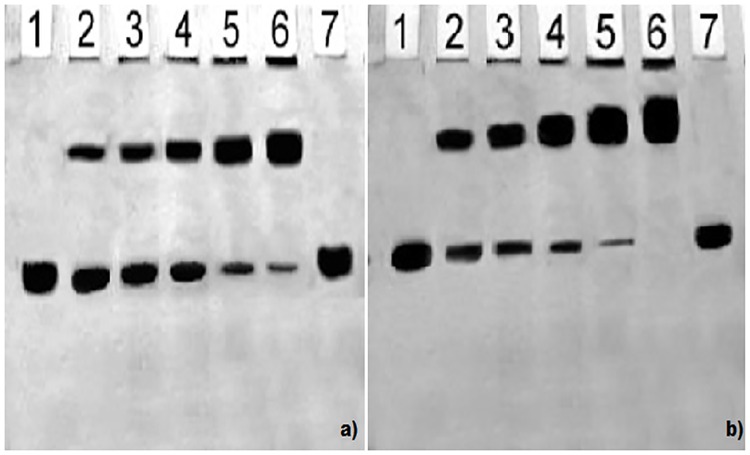
Gel retardation assay. SDS-PAGE of affinity purified IgG from sera of diabetespatients to N-IgG(a) and OH^•^-MG-IgG(b) on 6% SDS-PAGE at 80 V for 4 hours. 10 μg each ofN-IgG(a) and OH^•^-MG-IgG (b) was incubated with 0, 10, 20, 30, 40 and 50 μg of affinity purified IgG (lane 1–6). Lane 7 contains IgG alone.

Gel retardation studies showed a slight mobility shift when N-IgG (used as inhibitor) was incubated with increasing concentration of IgGfrom diabetes patient (serum sample #12). However, incubation of OH^•^-MG-IgG with IgGfrom diabetes patient resulted in a retarded mobility and proportional increase in the formation of high molecular weight immune complexes. Also, increase in molecular mass prevented the complete penetration of sample into the gel and its retention was observed at the origin of well (Fig 12).

## Discussion

T2DM is a very complex and multifactorial metabolic disease characterized by insulin resistance and β cell failure that consequently leads tohyperglycemia[42]. The role of hyperglycemia has been the central focus in the understanding of diabetic complications that have potentially affected the life quality and expectancy and has assumed a state of huge financial burden on the global healthcare[43].It has become imperative to understand the different perspective of the causes of diabetes type 2 so as to understand its pathogenesis. Furthermore, the role of ROS,generated during various metabolic events in vivo, is believed to play a key role in pathological processes observed in T2DM[44]. This study has aimed towards understanding the cumulative effect of OH^•^and MG on the structural integrity of IgG and its effect on the generation of autoimmune response in T2DM.IgG was modified separately by OH^•^ and MG, and the modification of MG-IgG by OH^•^ was also studied. The results indicate an extensive damage in the IgGwhen modified by OH^•^ and MG as compared to the damage caused by both the modifying agents separately. The78.3% hyperchromicity in UV absorbance values forOH^•^-MG modifiedIgGas compared to 55.9% and 64.7% for OH^•^-IgG and MG-IgG samples, when compared with N-IgG, suggests a stronger structural damage of IgG by OH^•^-MG. IgG modified by OH^•^-MG reflect highly exposed chromophoric aromatic amino acid residues, indicating extensive structural perturbations in the molecule.Appearance of a hump like structure in MG-IgG and OH^•^-MG-IgG indicate the formation of AGEs. Similar changes in absorbance have been reported earlier[45, 46].Fluorescence spectroscopy is a valuable parameter to monitor microenvironment around aromatic amino acid residues [47]. Our results indicate quenching in both tyrosine and tryptophan fluorescence.The observed quenching may be explained due to modification/destruction in tyrosine and tryptophan microenviroment [48],in addition to the formation of aggregates. It is the burial of fluorophoric amino acids during aggregation that causes quenching[49–52].Glycoxidised samples showing highest quenching followed by MG and OH^•^ treated IgG in comparison to N-IgG clearly indicate maximum microenvironment change and aggregate formation in OH^•^-MG-IgG followed by MG-IgG and OH^•^-IgG in comparison to the native protein. Another fluorescence spectralcharacterstic of AGE formation was observed in MG-IgG and OH^•^-MG-IgG when the samples were excited at 370 nm with emission at 435 nm. Increase in ANS fluorescence intensity indicates reorganisation in protein conformation leading to exposure of hydrophobic patches and thus making the sample more accessible to ANS fluorophore[53, 54]. Maximum unmasking of hydrophobic residues was observed in glycoxidised samples (OH^•^-MG-IgG). Glycated (MG-IgG) and hydroxylated (OH^•^-IgG) controls were comparatively less altered as can be observed from ANS fluorescence spectra. ANS fluorescence was lacking in N-IgGsample. Changes in carbonyl content, a prominent oxidation marker,was also studied. Accumulation of protein carbonyl owing to glycoxidation reactions is associated with various pathologies like diabetes and its associated complications, adult respiratory syndrome, pulmonary fibrosis, rheumatoid arthritis, parkinson’s disease, alzheimer’s disease, cystic fibrosis etc. The increase is due to oxidation of arginine, lysine, proline, histidine and other amino acid residues [55, 56]. We observed a large increase in carbonyl content in OH^•^-MG modified samples followed by MG and OH^•^ treated samples, in comparison to N-IgG. The results indicate an aggressive glycoxidative damage caused by the combined action of MG and OH^•^ than their individual effects. In vivo accumulation of these carbonyls results in carbonyl stress that alters protein activity. Similarly, 79.0%, 61.0% and 47.0% decrease in free sulphydryl group content in the OH^•^-MG-IgG, MG-IgG and OH^•^-IgG as compared to N-IgG, indicates oxidative modifications in IgG[56, 57]. Mass spectrometry is an accurate and robust tool that provides absolute and fast analysis of molecular mass of sample. To validate the attachment of OH^•^ residues in terms of accuracy and increment of molecular weight, MALDI-TOF analysis was performed. The observed chromatograms (both native and modified counterparts) had 2 peaks corresponding to signals obtained from single and double charges on protein molecule owing to ionization. Hydroxylation, glycation and glycoxidation were directly evident in the form of signature 194.6, 921.8 and 1110.1 Da mass shifts in spectra respectively. The observed increase in m/z values of modified IgG samples compared to native counterpart is due to attachment of MG and/or OH^•^groups. The number of MG and/or OH^•^ residues that have been linked to IgGmolecule was calculated considering the condensation of 1 MG and 1 OH^•^ residue may increase the mass by 72[58] and 17 Da [59]respectively. The results showed the attachment of 11 OH^•^ groups to OH^•^-IgG, 13 MG groups to MG-IgG and same number of OH^•^ and MG groups to OH^•^-MG-IgG. This attachment of MG and OH^•^ groups affect conformation/structure of IgG that lead to impairment of its functions, with maximum alteration in OH^•^-MG-IgG[58, 60]. Aggregation and crosslinking in protein are among the major biochemical changes in pathologies like diabetes mellitus and ageing. Both MG (lysine-lysine, lysine-arginine) and OH^•^ (dityrosine) are known to form covalently crosslinked aggregates[61, 62]. In our results N-IgGappeared like uniformly distributed granules. However, post MG and OH^•^ treatment the IgG appeared as red colored rough and irregular spots of variable sizes spread non-uniformly over the background. Highly crosslinked and entangled aggregates were observed in glycoxidisedIgG (OH^•^-MG-IgG). The alterations in the micro-architectural details were further confirmed by SEM. The images were visualized at a resolution of 1000X. Micrograph ofN-IgGappeared to have granular structures distributed uniformly over the surface. Unlike native, MG and OH^•^ modified samples were found to have irregular rod shaped structures of variable length aligned parallel and perpendicular to each other. The glycoxidisedIgG presented a highly dense micrograph more like that of fibrils. It may be concluded that aggregation in IgG is inextricably linked to conformational changes brought about by MG and OH^•^. The two in combination are highly potent to initiate a series of processes that ultimately lead to aggregation of IgG. Accumulation of these adducts/crosslinks/aggregates form the basis of a variety of pathologies such as renal damage, cataract, alzheimer’s disease, hypertension and diabetes [63–67]. Several proteins like fibrinogen, hemoglobin, LDL, collagen, serum albumin, β-2 microglobulin have been reported to form aggregates post modification [68–70]. Antioxidant activity of non-enzyme proteins is widely known. The activity is imparted by amino acids such as cysteine, tryptophan, histidine, methionine and tyrosine. It has been reported that blood proteins contribute 10–50% of peroxyl radical scavenging activity of plasma [40, 71–73]. Effect of modifications on the antioxidant property of IgG was checked by free radical induced haemolysis assay. OH^•^-MG-IgG showed significantly reduced antioxidant activity followed by glycated and hydroxylated samples.N-IgG protected AAPH induced RBC haemolysis to the maximum extent and thus exhibited highest antioxidant activity. The decreased antioxidant activity in the modified IgG samples is due to ligand induced modification of amino acid side chains. Similar results have been reported earlier also [33]. In order to evaluate the role of structural modifications of IgG on its immunogenicity, we studied the generation of neo-epitopes upon modification of IgG,that could potentially lead to an aggressive autoimmune response in T2DM. The results indicate highly immunogenic nature of OH^•^-MG-IgG compared to that of MG-IgG, OH^•^-IgG andN-IgG. The competitive inhibition data showing substantially higher inhibition of diabetes auto-antibodies activity by OH^•^-MG-IgGexplains high specificity of these antibodies towards the modified epitopes on IgG, compared to that of glycated, hydroxylated andN-IgGsamples. The higher recognition of OH^•^-MG-IgG by the auto-antibodiesfrom diabetes patients point towards epitope sharing between in vitro modified IgG and the the immunogenic epitopes that appear in diabetes patients. The solid phase immunoassay results were further confirmed by gel shift assay wherein incubation of OH^•^-MG-IgG,with increasing concentrations of the antibodies from diabetes patients, resulted in a retarded mobility and proportional increase in the formation of high molecular weight immune complexes. The results indicate the generation of auto-antibodies in diabetes patients against the modified epitopes of IgG.

## Conclusion

From the above results we conclude that the damaging potential of glycoxidation is quite high as compared to glycation or oxidation alone. Glycoxidation is the result of glycation and generation of ROS (i.e. oxidative stress); both factors are dominant in diabetes. Appreciable recognition of the glycoxidatively modified epitopes by the antibodies of diabetes patients may be used as a parameter of disease progression.
